# Supplementary material for: Public Awareness of Chronic Kidney Disease in Jazan Province, Saudi Arabia—A Cross-Sectional Survey
Source: Healthcare (Basel). 2022 Jul 25;10(8):1377. doi: 10.3390/healthcare10081377 (PMC9330694; doi:10.3390/healthcare10081377)
Supplement: Supplementary file 1 [file healthcare-10-01377-s001.zip › healthcare-1758845-supplementary.pdf]

Table S1. Knowledge of kidney problems and CKD.

Questions	Responses Yes n (%)	No / I don't know n (%)
1. Many people are living with kidney problems.	259 (58.9)a	181 (41.1)
2. The role of kidney in the body is to filter out waste and water from the blood.	336 (76.4)a	104 (23.6)
3. Kidney filters 200 quarts blood per day.	196 (44.5)	244 (55.5)b
4. Kidney maintains blood pressure.	278 (63.1)a	162 (36.8)
5. Kidney maintains blood pH.	289 (65.7)a	151 (34.3)
6. Kidney maintains minerals of the body.	302 (68.6)a	138 (31.4)
7. Nephron is the fundamental unit of the kidney.	257 (58.4)a	183 (41.6)
8. Each adult human kidney contains roughly one million nephrons.	198 (45)	242 (55) b
9. Urine is formed in three-step process: Glomerular filtration, tubular reabsorption, and tubular secretion.	312 (70.9)a	128 (29.1)
10. CKD leads to an increase poisonous substances in the body.	340 (77.3)a	100 (22.7)
11. An increase in creatinine is always indicative of kidney problems.	190 (43.2)	250 (56.8)b
12. Back pain is an indication of kidney problem.	256 (58.2)a	184 (41.8)
13. CKD can cause retention of fluid.	312 (70.9)a	128 (29.1)
14. Anyone of any age can get kidney diseases.	320 (72.7)a	120 (27.3)
15. An individual can survive with one kidney.	346 (78.6)a	94 (21.4)
16. Most cases of CKD in adults are inherited rather than acquired.	146 (33.2)	294 (66.8)b
17. Every 14 days a new name is added to the kidney transplant waiting list.	124 (28.2)	316 (71.8)b
18. Kidney diseases are preventable.	349 (79.3)a	91 (20.7)

a - Answers with high proportion of correct responses; b - Answers with high proportion of incorrect responses

Table S2. Awareness of risk factors associated with CKD

Questions	Responses Yes n (%)	No / I don't know n (%)
1. Diabetes is the risk-factor for CKD.	293 (66.6)a	147 (33.4)
2. Hypertension is the risk-factor for CKD.	307 (69.8)a	133 (30.2)
3. Abuse of pain reliever drugs (NSAIDs)can cause CKD.	333 (75.7)a	107 (24.3)
4. Dehydration can cause an acute kidney Injury (AKI).	330 (75.0)a	110 (25)
5. Infections can cause CKD.	206 (46.8)	234 (53.2)b
6. Dye used in CAT and CT scans can cause CKD.	226 (51.4)a	214 (48.6)
7. Frequent use of enemas can cause CKD.	85 (19.3)	355 (80.7)b
8. Eating too much red meat can cause CKD.	97 (22)	343 (78)b
9. Blood volume depletion can cause CKD.	176 (40)	264 (60)b

10. Drinking alcohol can cause CKD.	322 (73.2)a	118 (26.8)
11. Smoking can cause CKD.	249 (56.6)a	191 (43.4)
12. Khat chewing can cause CKD.	92 (20.9)	348 (79.1)b
13. HIV is a risk factor for CKD.	213 (48.4)	227 (51.6)b
14. Hepatitis is a risk factor for CKD.	188 (42.7)	252 (57.3)a
15. Autoimmune disease are risk factor for CKD.	173 (39.3)	267 (60.7)b
16. Heart diseases are risk factor for CKD.	205 (46.6)	235 (53.4)b
17. People over the age of 60 rears are at risk for CKD.	250 (56.8)a	190 (43.2)
18. Frequent micturition is bad for your kidney.	99 (22.5)	341 (77.5)
19. Urinary tract obstruction or dysfunction and tubulo-interstitial disease are both recognized causes of CKD.	294 (66.82)a	146 (33.2)
20. Hypokalemia is more commonly associated with the development of CKD.	236 (53.6)a	204 (46.4)
21. Vascular disease is the cause for CKD.	184 (41.8)	256 (58.2)b
22. Recurrent kidney stones is a cause of CKD.	299 (68)a	141 (32)
23. Herbal medicine can cause CKD.	353 (80.2)a	87 (19.8)
24. Glomerulonephritis is a risk factor for CKD.	325 (73.9)a	115 (26.1)
25. Statins use is a risk factor for CKD.	89 (20.2)	351 (79.8)b

a - Answers with high proportion of correct responses; b - Answers with high proportion of incorrect responses

Table S3. Awareness of complications associated with CKD.

**Questions**	**Responses**	
**Awareness questions**	**Yes n (%)**	**No / I don't know n (%)**
1. Hyperkalemia is a complication associated with CKD.	174 (39.5)	266 (60.5)b
2. Uremia is a complication associated with CKD.	234 (53.2)a	206 (46.8)
3. Edema is a complication associated with CKD.	182 (41.4)	258 (58.6)b
4. Nausea and vomiting are complications associated with CKD.	172 (39.1)	268 (60.9)b
5. Coma is a complication associated with CKD.	144 (32.7)	296 (67.3)b
6. End-stage renal disease is more common in women than in men	101(23)	339 (77)b
7. Almost half of adult patients with CKD demonstrate depressive symptoms	177 (40.2)	263 (59.8)b
8. Anemia is a complication associated with CKD.	171(38.9)	269 (61.1)b
9. Bone disease and high phosphorous are the complications associated with CKD.	151(34.3)	289 (65.7)b
10. Fluid build-up is the complication associated with CKD.	286 (65.0)a	154 (35.0)
11. Angina pectoris is the complication associated with CKD.	80 (18.2)	360 (81.8)b
12. Worsening heart failure is a complication associated with CKD.	122 (27.7)	318 (72.3)b
13. Abnormalities in bone minerals are complications associated with CKD.	138 (31.4)	302 (68.6)b
14. Loss of appetite is a complication associated with CKD.	212 (48.2)	228 (51.8)b
15. Fatigue is a complication associated with CKD.	146 (33.2)	294 (66.8)b
16. Sleep disturbance is a complication associated with CKD.	85 (19.3)	355 (80.7)b

17. Pruritis is a complication associated with CKD.	73 (16.6)	367 (83.4)[b]
18. Infertility is a complication associated with CKD.	91 (20.7)	349 (79.3)[b]
19. Coagulopathy is a complication associated with CKD.	80 (18.2)	360 (81.8)[b]
20. Neuropathy is a complication associated with CKD.	183 (41.6)	257 (58.4)[b]

a - Answers with high proportion of correct responses; b - Answers with high proportion of incorrect responses
